# Labour companionship and respectful treatment of women during childbirth: a cross-sectional study across 16 hospitals in Benin, Malawi, Tanzania and Uganda

**DOI:** 10.1136/bmjph-2024-002462

**Published:** 2025-05-12

**Authors:** Soha El-Halabi, Kristi Sidney Annerstedt, Christian Agossou, Fadhlun M Alwy Al-Beity, Andrea B Pembe, Phillip Wanduru, Bianca Kandeya, Jean-Paul Dossou, Helle Molsted-Alvesson, Meghan A Bohren, Claudia Hanson

**Affiliations:** 1 Department of Global Public Health, Karolinska Institutet, Stockholm, Sweden; 2 Centre de Recherche en Reproduction Humaine et en Démographie (CERRHUD), Cotonou, Benin; 3 Department of Obstetrics and Gynaecology, Muhimbili University of Health and Allied Sciences, Dar es Salaam, Tanzania, United Republic of; 4 Department of Health Policy, Planning, and Management, Makerere University, Kampala, Uganda; 5 Center for Reproductive Health, Kamuzu University of Health Sciences, Blantyre, Malawi; 6 Gender and Women’s Health Unit, Nossal Institute for Global Health, School of Population and Global Health, University of Melbourne, Melbourne, Parkville, Australia; 7 Department of Disease Control, London School of Hygiene & Tropical Medicine, London, UK; 8 Centre of Excellence in Women and Child Health, Aga Khan University, Nairobi, Kenya

**Keywords:** Cross-Sectional Studies, Epidemiology, Public Health Practice

## Abstract

**Introduction:**

Evidence suggests that women value and benefit from having a labour companion during childbirth. However, the applicability of the evidence to low-income and lower-middle-income countries is limited and varies. Thus, we assessed the association between the presence of labour companions and mistreatment in 16 hospitals of Benin, Malawi, Tanzania and Uganda.

**Methods:**

We conducted a cross-sectional survey using a validated questionnaire administered to women at discharge after birth between December 2021 and March 2024. The main outcomes were factor-weighted respectful treatment score and its subscores on maintained respect and dignity, privacy and maintained confidentiality and no physical and verbal abuse. The independent variable was labour companionship. We assessed the association between labour companionship and mistreatment using a linear regression model with fixed effects and cluster robust standard errors.

**Results:**

Of the 4006 included women, 39% (n=1573) had a companion during labour and/or birth. Women across the four countries and regardless of companions’ presence reported high degrees of maintained privacy and confidentiality (subscores ranging between 9.5/10 in Benin to 9.9/10 in Malawi). The presence of a labour companion was significantly associated with the absence of physical and verbal abuse experiences (β=0.07; p=0.004; 95% CI: 0.02; 0.12).

**Conclusions:**

Our study adds evidence on the positive relation between labour companionship and physical and verbal abuse. The coverage of labour companionship was low across the four countries. We call for the implementation of labour companionship to allow for greater benefits of this practice.

**WHAT IS ALREADY KNOWN ON THIS TOPIC**

- A Cochrane review has shown that the presence of a labour companion during labour and birth enhances women’s birth experiences and has several benefits to the woman and the baby.
- Little is known about the association between labour companionship and mistreatment during childbirth in Benin, Malawi, Tanzania and Uganda.

**WHAT THIS STUDY ADDS**

- Our results show that the presence of a labour companion is significantly associated with the reduction of physical and verbal abuse experiences.
- Companionship coverage was low (39%) across the four countries, and women were not allowed to have companions continuously during labour and birth.

**HOW THIS STUDY MIGHT AFFECT RESEARCH, PRACTICE OR POLICY**

- We call for a systematic implementation of labour companionship, including the development of supportive policies to allow for a greater benefit of this practice.

## Background

 Mistreatment of women during labour and childbirth is a global phenomenon experienced by women across different healthcare settings.^1^ Types of mistreatment include physical and verbal abuse, stigma, neglect, breaches of confidentiality and privacy, detainment in health facilities and lack of supportive care.^1^ Labour observations and community-based surveys in four low-income and-middle-income countries showed that mistreatment of women is highest during the 30 minutes before birth and until 15 minutes after.^2^ Younger and less educated women in lower wealth groups are at a higher risk for mistreatment.^2^,^5^ This is not only a violation of their human rights and autonomy^6^ but may also jeopardise the health of both the woman and the baby and influence future health-seeking behaviours.^7 8^

Labour companionship, for women who desire it, is a recommended evidence-based intervention to enhance women’s childbirth experiences and has been recognised as a key component of respectful maternity care.^9^,^11^ Labour companionship is defined as the presence of a person of the woman’s choice such as a partner, family member, friend or a doula throughout the childbirth experience.^12^ Most women prefer a labour companion to be present throughout the childbirth process. Moreover, there are proven benefits for both the mother and baby,^13 14^ including shorter duration of labour and decreases in caesarean section, instrumental vaginal birth and intrapartum analgesia, together with improved fetal outcomes.^12^

Labour companions play an essential role in advocating for women’s rights and well-being during childbirth and are considered as witnesses to the process of childbirth,^15 16^ which may in return reduce the risk of mistreatment.^17 18^ Evidence from lower-middle-income settings suggests that women accompanied by a labour companion during childbirth are less likely to report physical and verbal abuse, and non-consented care.^19^ Women report higher levels of satisfaction with childbirth care and birth experiences.^20^,^25^ Moreover, the presence of a labour companion may decrease levels of childbirth fear and enhance self-efficacy among women.^26^

In view of the evidence of a positive effect of labour companionship on mistreatment, the Action Leveraging Evidence to reduce perinatal Mortality and morbidity in Sub-Saharan Africa (ALERT) study promoted labour companionship, among other interventions, to improve responsiveness during labour and birth. ALERT was implemented as a stepped-wedged trial in 16 hospitals in Benin, Malawi, Tanzania and Uganda. Details on the intervention and the evaluation are available elsewhere.^27 28^ The evaluation of the intervention included the development and validation of a questionnaire to measure mistreatment and responsiveness as reported by women discharged from giving birth in these hospitals.^27 28^

To date, existing studies have quantified the prevalence of mistreatment during childbirth across countries^129^,^34^ and identified associated factors.^5 31^ Several scholars have also explored and identified preferences for labour companionship among women and healthcare providers.^1415 17 18 35^,^37^ However, there is limited evidence on the relation between labour companionship and respectful treatment during childbirth in Benin, Malawi, Tanzania and Uganda using validated tools. To better understand the potential positive relation between labour companionship and mistreatment, we aimed to assess the associations between labour companions and respectful treatment as reported by women in Benin, Malawi, Tanzania and Uganda. We hypothesised that women who were accompanied by a labour companion would experience less mistreatment compared with those who gave birth without a companion.

## Material and methods

This was a cross-sectional study in 16 hospitals in Malawi, Benin, Tanzania and Uganda (table 1). We followed the Strengthening the Reporting of Observational Studies in Epidemiology to report on this study (online supplemental additional file 1).

**Table 1:** Descriptive sociodemographic characteristics of 4006 women in Benin, Malawi, Tanzania and Uganda

	BeninN=1003n (%)	MalawiN=1004n (%)	TanzaniaN=996n (%)	UgandaN=1003n (%)	TotalN=4006n (%)
Age mean (SD)	27.3 (6.1)	24.1 (6.3)	26.7 (6.9)	25.1 (6.0)	25.1 (6.4)
Education level completed N=3684					
Primary	266 (32.4)	684 (70.5)	564 (60.6)	401 (41.6)	1915 (52.0)
Secondary	445 (54.3)	270 (27.8)	311 (33.4)	500 (51.9)	1526 (41.4)
University	109 (13.3)	16 (1.6)	56 (6.0)	62 (6.4)	243 (6.6)
Currently employed					
	734 (73.2)	591 (58.9)	598 (60.0)	554 (55.2)	2477 (61.8)
Number of previous pregnancies median (Q1; Q3)					
	3 (1; 4)	2 (1; 3)	2 (1; 3)	2 (1; 4)	2 (1; 4)
Number of previous births N=3940 median (Q1; Q3)					
	2 (1; 4)	2 (1; 3)	2 (1; 3)	2 (1; 3)	2 (1; 3)
Mode of birth*					
Vaginal†	540 (53.8)	852 (84.9)	684 (68.7)	684 (68.1)	2760 (68.9)
Caesarean section	463 (46.2)	152 (15.2)	312 (31.3)	320 (31.9)	1247 (31.1)
Outcome of birth* N=3999					
Alive	958 (95.5)	971 (97.4)	977 (98.1)	964 (96.1)	3870 (96.8)
Stillbirth	45 (4.5)	26 (2.6)	19 (1.9)	39 (3.9)	129 (3.2)

* During last pregnancy.

† Includes vacuum extraction, forceps and assisted breech.

### Study setting

Study countries were a mix of low-income and lower-middle-income countries according to the World Bank classification^38^ and were situated in East and West Africa. All countries had a low nurse/midwife per 1000 people ratio, with the highest being in Uganda (1.6 per 1000 people).^39^ The maternal mortality ratio per 100 000 live births was lowest in Tanzania (238; uncertainty interval: 174–381) and highest in Benin (523; uncertainty interval: 397–768).^40^ Study hospitals included public (n=3 per country) and faith-based (n=1 per country). The selection of hospitals was purposive and is described in detail elsewhere.^28^

### Study tool

The hospital-based cross-sectional survey was designed by integrating two pre-existing tools from Bohren *et al* on mistreatment^41^ and Afulani *et al* on person-centred maternity care.^4^ Further details are described elsewhere.^27^ The aim of the tool was to provide data for three secondary outcomes for the ALERT trial: (1) system and healthcare provider’s responsiveness to women’s needs during childbirth, (2) manifestations of mistreatment during labour and birth and (3) breastfeeding practices. It was administered to women during the postpartum period, immediately before discharge from hospitals (24 hours in case of vaginal birth and between 48 and 72 hours in case of caesarean section). The tool had 12 sections which included questions on women’s demographic characteristics, childbirth incurred costs, verbal and physical abuse, social support, communication with healthcare providers, overall satisfaction, hospital environment and newborn responsiveness. The tool was translated and administered in local languages, that is, Chichewa in Malawi, French in Benin, Luganda in Uganda and Kiswahili in Tanzania. Translations were done and reviewed by the research teams in the four countries.^42^

The questionnaire was validated through performing a psychometric analysis on data collected from Benin, Malawi, Tanzania and Uganda as part of the ALERT intervention.^27^ This resulted in the development of factor-weighted scores on respectful treatment, responsiveness and an overall score combining both domains. The respectful treatment score included three subscales on maintained respect and dignity, maintained privacy and confidentiality and lack of physical and verbal abuse. The responsiveness score included subscales on communication and supportive care, hospital environment and social support. This study uses a subset of the items in the validated questionnaire with a focus on respectful treatment score and its subscores. The subscores with included items and scales are provided as online supplemental additional file 2.

We used formative research data from a health facility assessment conducted as part of the ALERT intervention to report on the available number of rooms and beds during labour and birth and hospital policies on companionship.

### Data collection

Data collection was ongoing in parallel to the stepped-wedged trial in the 16 hospitals. The study tool was administered by trained data collectors (11 women and 3 men) through tablet-based software, Research Electronic Data Capture (REDCap) hosted at Karolinska Institutet, Sweden.^43 44^ Data collectors had backgrounds in nursing or social sciences. An initial training was conducted for all data collectors on tool administration and to cross-check translations. This was followed by refresher training sessions prior to the start of each data collection round.

The questionnaire was administered to 50 women per study hospital every 6 months from December 2021 to March 2024 (online supplemental additional 3). Sample size was estimated as part of the ALERT intervention using 50 interviews per cluster across 16 clusters (hospitals), to indicate an improvement of 75%, assuming a baseline prevalence of 4%, an intraclass correlation coefficient of 0.1, a power of 77% and an anticipated outcome value of 1%.^28^ A maximum of 14 women were targeted for recruitment per day. Data collectors applied a randomisation factor to recruit the target number in each hospital. The randomisation factor was based on the number of eligible women being discharged from the study hospitals. For example, hospitals with (1) fewer than 14 women being discharged per day, all eligible women were approached, (2) 20–29 women being discharged per day, every other woman was approached (randomisation factor=1:2) and (3) more than 30 women being discharged per day, every third was approached (randomisation factor=1:3).

### Participants

The inclusion criteria included (1) all women who gave birth at the study hospitals to a baby weighing more than 1000 grams or had a gestational age of more than 28 weeks, (2) providing written consent to participate and (3) agreeing to link their data to ALERT’s perinatal e-Registry (an electronic registry set up as part of the ALERT intervention to document all births at the study hospitals).^45^

### Variables

The outcomes were factor-weighted scores expressed as ‘respectful treatment score’ and reported as mean percentage of the best practice with 100% being the optimal practice. The respectful treatment score included three subscales (1) maintained privacy and confidentiality, (2) maintained respect and dignity and (3) no physical and verbal abuse. The scores ranged from 0 to 10. A higher score for the respectful treatment score meant fewer mistreatment experiences as reported by the women.^27^

The independent variable was labour companion with the women giving birth. The variable was coded as two levels with ‘yes’ and ‘no’. We considered that a woman had a labour companion if she had answered yes to the presence of a labour companion during labour and/or birth. We did not include observations of women who had answered ‘did not want anyone’ for the item ‘were you allowed to have a companion during labour/birth’. This decision was made because the proportions of women who preferred not to have a companion varied across groups, and the sample size was insufficient for a regression analysis. Additionally, we could not methodologically group these observations with ‘no’ since we acknowledged a difference between a woman not having a companion and a woman not wanting one. Survey data were merged with data from the perinatal e-registry to describe maternal age and mode of birth.

### Statistical analyses

All data were imported from REDCap to Stata V.16^46^ for data cleaning and analysis. We used descriptive statistics to report on age, parity, educational attainment, mode of birth and birth outcome. We analysed characteristics of labour companionship, including when they were allowed to be with the woman (labour/birth). Person of choice was considered a proxy for labour companion who was with the woman in the maternity ward (either labour, birth or both). We used descriptive statistics to report any form of mistreatment by companionship status (presence/absence). We conducted the analysis on a country level to compare differences across the four countries.

We assessed the associations between labour companionship presence and mistreatment using a linear regression model with fixed effects and cluster robust standard errors. The linear regression model with fixed effects enabled us to estimate a model that adjusted for all covariates shared by independent observations within each cluster (eg, hospital). We treated age, parity, educational level and mode of birth as potential confounders based on previous research.^2 4^ Parity was treated as a dichotomous variable, first versus more than one previous pregnancy. This was based on our assumption that the experiences reported by women might differ among those who are giving birth for the first time versus those who have already given birth at least once. We did not differentiate between in-labour caesarean section and prelabour caesarean section. Additionally, given that the ALERT intervention targeted companionship, we adjusted for the intervention in line with the stepped-wedge trial roll-out. The distribution of the outcome was non-parametric, so we applied cluster robust standard errors to adjust for model misspecification. Results were presented using beta (ß) coefficients, 95% CIs and p values with p<0.05 to be statistically significant.

## Results

**Figure 1:**
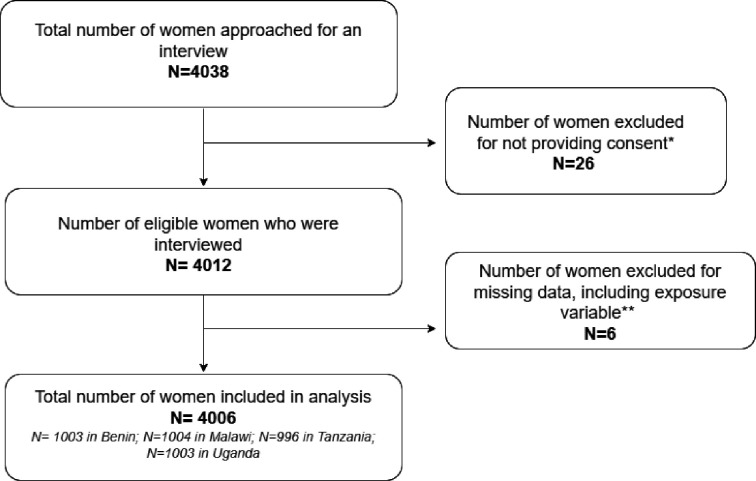
Flow chart of total number of women included in analysis. *Includes consent to participate in survey or consent to link data to perinatal e-registry; **Missing values for all study variables, including one observation with missing response for exposure variable.

We recruited a total of 4038 women across the four countries. We excluded 26 women who did not consent to participate, 5 women with missing values for all study variables in the record and 1 woman for missing response on the exposure variable. After excluding these observations, the responses of 4006 women; 1003 from Benin, 1004 from Malawi, 996 from Tanzania and 1003 from Uganda were included in our analysis. Figure 1 illustrates the total number of women included and excluded in analysis.

Out of the 4006 women included in our analysis, the mean age ranged between 24 years in Malawi and 27 years in Benin. Vaginal birth was the most common mode of birth across countries (n=2760; 68.9%). Women had a median of two previous pregnancies across all countries.

### Labour companionship practices across the four countries

All hospitals had a high case load, limited space and strict hospital policies on labour companionship, with some variations. Benin had the least number of delivery rooms (one). Only two hospitals in Malawi used separators in their labour rooms. On the contrary, labour rooms in Uganda did not include separators, and only two hospitals used separators during birth.

There were wide country variations in companionship coverage during labour or birth, ranging from 4.7% (47/996) in Tanzania to 96.7% (971/1004) in Uganda. However, this proportion decreased to 1.2% (n=12) in Benin and Tanzania and 69% (690/1003) in Uganda for women who had a companion in labour and birth. A total of 16% (162/1003) of women in Benin did not want to be accompanied by anyone during labour and/or birth. Our subanalysis presents minor changes in labour companionship coverage across the data collection rounds, except for Malawi (online supplemental additional 4). Hospital policies in all countries did not allow companions to be present with women during birth (ie, in delivery rooms). In Benin, women were only allowed to be with their companions during the postpartum period; however, a female companion was called in case of complications during labour. Only one hospital in Tanzania allowed companions to be present during labour as of January 2023, after the implementation of a labour companionship model as part of the ALERT intervention. Female relatives in Tanzania were allowed during post partum in case the woman had given birth through caesarean section or had experienced maternal complications; otherwise, they could be present with the woman during visiting hours only. In Uganda, labour companions were allowed to accompany the woman outside the maternity ward during labour only, due to the crowded and limited physical space (table 2).

**Table 2:** Characteristics of maternity wards in the four countries

	BeninN=1003	MalawiN=1004	TanzaniaN=996	UgandaN=1003
Monthly # of births*	201 (146; 507)	431 (325; 531)	153 (114; 227)	368 (213; 508)
Average # of labour rooms†	1	2	1	2
Average # of beds in labour room†	3	3	6	3
Separators used in labour rooms	No	In 2 hospitals	No	No
Average # of delivery rooms†	1	5	3	2
Average # of beds in delivery room†	5	6	4	4
Separators used in delivery room	Yes	Yes	Yes	In 2 hospitals
Woman had a companion				
Yes—labour only	78 (7.8)	126 (12.6)	31 (3.1)	265 (26.4)
Yes—birth only	8 (0.8)	38 (3.8)	4 (0.4)	16 (1.6)
Yes—labour and birth	12 (1.2)	292 (29.1)	12 (1.2)	690 (68.8)
No	743 (74.1)	534 (53.2)	829 (83.2)	28 (2.8)
Did not want‡	162 (16.2)	13 (1.3)	120 (12.0)	4 (0.4)
Hospital policy on allowing companions				
	Post partum	Labour and post partum	Labour§ and post partum¶	Labour and post partum
Type of companion allowed	Female relative	Partner in 1 hospital otherwise female relative	Female relative	Female relative

* Based on data from ALERT’s e-registry for the year 2023.

† Based on data from ALERT’s health facility assessments in the 16 hospitals.

‡ Proportion of women who did not want a companion in labour and birth.

§ Only allowed in one hospital where cubicles are available as of January 2023.

¶ Companions are only allowed to be present with women who had postpartum complications or undergone CS.

ALERT, Action Leveraging Evidence to reduce perinatal Mortality and morbidity in Sub-Saharan Africa; CS, caesarean section.

### Respectful treatment scores as reported by women across the 16 hospitals

The analysis of the associations between companionship and respectful treatment shows a diverse pattern. Women, in Benin, who were accompanied by a companion reported lower degrees of respectful treatment compared with those who did not have a companion (score of 83.9 vs 85.8 of 100). In Malawi, Tanzania and Uganda, scores were not different among women having or not having a companion. Women across the four countries and regardless of their companions’ presence perceived their physical privacy to be maintained and their information to be treated confidentially throughout labour and birth, with the subscore ranging from 9.5/10 in Benin to 9.9/10 in Malawi. In Tanzania, women who were accompanied by a companion reported significantly higher (p=0.04) scores on the subscore maintained respect and dignity compared with those who were not (9.3 vs 8.9/10). The subscore on no physical and verbal abuse was lowest compared with the other scores, with women who did not want a companion in Malawi and Tanzania scoring lowest (7.1/10) (table 3).

**Table 3:** Respectful treatment score and its subscores across the four countries by companionship availability

Companion present	Benin		Malawi		Tanzania		Uganda	
	YesN=98	NoN=743	YesN=543	NoN=457	YesN=47	NoN=829	YesN=971	NoN=28
Respectful treatment score*	**83.9** (**7.9**)	**85.8** (**6.5**)	87.4 (6.2)	86.8 (7.0)	87.6 (5.8)	85.7 (7.2)	86.3 (6.4)	86.7 (7.1)
Maintained respect and dignity	8.4 (1.6)	8.5 (1.4)	9.2 (1.5)	9.1 (1.6)	**9.3** (**0.8**)	**8.9** (**1.4**)	9.0 (0.9)	8.9 (1.1)
Maintained privacy and confidentiality	**9.5** (**1.1**)	**9.8** (**0.7**)	9.9 (0.7)	9.8 (1.0)	9.8 (1.5)	9.8 (1.1)	9.6 (1.5)	9.8 (0.7)
No physical and verbal abuse†	7.2 (1.1)	7.4 (1.0)	7.1 (0.7)	7.1 (0.5)	7.2 (0.5)	7.1 (0.8)	7.3 (0.9)	7.3 (1.3)

* Mean percentage ranges between 0 and 100.

† Score ranges between 0 and 10; in bold statistically significant differences between scores.

No, companion not present; Yes, companion present during labour and/or birth.

### Association between presence of a companion and respectful treatment score as reported by women

The presence of a companion was significantly associated with the lack of physical and verbal abuse (β=0.07; p=0.004; 95% CI: 0.02; 0.12). We observed a positive direction between the presence of a labour companion and the scores on respectful treatment and maintained respect and dignity, although not statistically significant (table 4).

**Table 4:** Crude and adjusted regression models

	Crude			Adjusted		
	β	P value	95% CI	β	P value	95% CI
Respectful treatment score	0.61	0.08	−0.08; 1.31	0.47	0.17	−0.23; 1.18
Maintained respect and dignity	0.13	0.06	−0.01; 0.26	0.07	0.51	−0.16; 0.32
Maintained privacy and confidentiality	−0.02	0.74	−0.13; 0.09	−0.01	0.80	−0.13; 0.10
No physical and verbal abuse	0.07	0.09	−0.01; 0.16	**0.07**	**0.004**	**0.02; 0.12**

Model is adjusted for age, education level, mode of birth, parity and interaction between hospital and intervention status (pre/post); 95% CI; reference was companion absent; values in bold are statistically significant.

## Discussion

This study assessed the association between the presence of labour companionship and respectful treatment as reported by women in 16 hospitals across Benin, Malawi, Tanzania and Uganda. Out of the 4006 women in the sample, 39% (n=1573) reported having a companion during labour and/or birth with wide variations between the countries. Women reported respectful treatment scores ranging between 83.9% in Benin and 86.3% in Uganda, indicating that a substantial proportion of women experience some form of mistreatment (around 17%). We found a significant association between the presence of a labour companion and the absence of verbal and physical abuse.

Our finding that only 39% of women had a labour companion is slightly higher than the proportion of a pooled rate (34%) of labour companionship from the sub-Saharan African region.^47^ The low levels^47 48^ are possibly due to restrictive policies and limited space as previously described.^35 49 50^ In Uganda, due to the crowded maternity wards, women who were in early labour stages were asked to stay outside. This gave them the opportunity to be with their labour companions. This might also explain the higher proportion of reported labour companionship in this study (96.7%) and the lower proportion (69%) of women who reported having a companion in labour and birth. Furthermore, while women were not permitted to have a companion at birth in Uganda, in practice they often had a companion in an intermittent manner for practical reasons. Therefore, we also consider labour companionship to be underimplemented in Uganda, despite the high proportions indicating otherwise. Similarly in Benin, the practice is underimplemented with a relatively low proportion of women allowed to have a companion in labour and birth.

Companions in the four settings had practical roles mostly related to preparing food, laundry and buying items and medication. Thus, their role in providing emotional support or being advocates for the women during labour and birth is limited. All four countries had limited physical space in their maternity wards and lacked training and supportive guiding policies to healthcare providers to implement the practice. When allowed to be present, most women were accompanied by a female relative. This was also reported by other studies from Tanzania and South Africa, due to social norms and limited privacy in the maternity ward.^22 36^

Women across the four countries reported relatively high degrees of respectful treatment, particularly for the subscore on maintained privacy and confidentiality. This finding is contradictory to evidence from other studies,^30 32^ where fear of privacy violation was among the key barriers to the implementation of labour companionship practices.^15 19^ We speculate that the higher values in our study may have been linked to the normalisation of some forms of abuse during childbirth by many women,^51 52^ particularly the no physical and verbal abuse subscore in this study. Adinew *et al* show that women tend to perceive some forms of abuse and disrespect as acceptable, especially if intended to save a life or to discipline "disobedient" women.^53^ This is also expressed by Khalil *et al*, indicating the normalisation of abuse within the sociocultural norms across countries in the Eastern Mediterranean Region.^54^ Self-reports on childbirth experiences can be very subjective to women’s expectations of care and behaviours they deem acceptable. Thus, the high reporting of respectful treatment might be a representation of women’s perception of having received the care that they expected to receive within these hospitals.

Women’s experiences of respectful treatment during childbirth remain less than optimal as reported in this study, which is concerning. Women expressed feelings of being neglected and having poor communication with healthcare providers in qualitative interviews conducted as part of the ALERT co-design intervention.^55^ Mistreatment of women during childbirth is a violation of their fundamental human rights^56^ and could influence their future health-seeking behaviours for maternal health services. More broadly, mistreatment of women during childbirth is an indicator of poor quality of care and may also be a reflection of the health system’s role in perpetuating mistreatment.^57^ The reported types of mistreatment in this study suggest an imbalance in power dynamics between the healthcare providers and the women and may be linked to several factors.^51 56^ The lower scores on physical and verbal abuse compared with the other subscores reflect the breakdown in respect and control exerted by healthcare providers as highlighted by other studies.^53 58 59^ This can also be driven by organisational factors that health workers endure, including shortages in staff, limited resources, high birth loads and limited accountability mechanisms.^1 60 61^

Our adjusted model showed a significant association between the presence of labour companions and lack of physical and verbal abuse. This finding is in line with the literature on labour companionship and mistreatment during childbirth.^1922 62^,^64^ These results may be explained by the implementation of the intervention in the four countries. As part of the ALERT intervention, the country teams in Malawi and Tanzania co-designed a companionship module in the hospitals, while targeting issues on respectful maternity care in the maternity ward. Some changes to the physical structure of the maternity ward were introduced in Malawi, including curtains for more privacy and the building of private rooms for birth which allowed the presence of a companion.^65^

### Strengths and limitations

The main strength of the analysis lies in the use of a single validated questionnaire across the 16 hospitals in Benin, Malawi, Tanzania and Uganda. This allowed us to describe the degrees of mistreatment while drawing comparisons across countries. We collected data on when companions were present with the woman (ie, labour and/or birth), which is often a missed variable in other studies.^19 48^

Social desirability bias needs to be considered as data were collected in hospitals before discharge. Validation studies have shown that they underestimate prevalences of mistreatment compared with studies done 1 month after discharge at home.^4 66^ Women may feel less secure in the hospital settings to report on mistreatment due to fear of poor treatment when seeking future care. Another potential explanation is that women had limited time to reflect on the birthing experiences (between 24 and 72 hours after birth), and they may have needed more time and distance to the event to reflect on mistreatment and abuse. To reduce the bias, interviews were carried out in a private room at the hospital premises and by data collectors who were identified as external to the staff, except in Uganda where data collectors were employed by the hospital. We acknowledge that our study may under-report mistreatment and abuse. However, postpartum surveys are a cost-effective strategy to obtain estimates, which is why we opted for this assessment method.

Our analysis was not stratified by mode of birth, and we are not sure of the practices on presence of a labour companion in the case of caesarean section. To our knowledge, hospital policies do not allow women to have a labour companion during elective caesarean sections. Women can remain with their companions during labour only in case of emergency c-sections. Similarly, our dominant outcome of birth was alive babies. We are not able to distinguish the differences in women’s perception of care based on the outcome of birth, given that a little over 2% of women across the four countries had a stillbirth. Future studies may benefit from subgroup analysis to bridge the knowledge gap on labour companionship and mistreatment of women who give birth through caesarean section or experience a stillbirth.

### Impact on practice and research

Our analysis presenting a statistically significant association between the presence of a companion and lack of verbal and physical abuse indicates potential benefits of this practice. The introduction and institutionalisation of companionship bears opportunities to reduce the different forms of mistreatment in labour wards. However, to have an effect, a more systemic implementation may be needed. Thus, it is important that guidelines on implementation are adapted to fit their contexts with training to support healthcare providers. At best, the maternity ward would require some changes to accommodate companions, such as the addition of chairs and curtains.

The literature highlights several data collection methods to quantify the burden of mistreatment during childbirth.^2 30 66^ However, there remains a methodological gap in the absence of a standardised tool which measures both respectfulness and mistreatment. More methodological work is also needed to develop a cost-effective data collection method which can be implemented in settings with limited resources. Future efforts are needed to quantify labour companionship coverage^48^ and mistreatment during childbirth, through a standardised tool, allowing for comparisons across studies in the different contexts.

## Conclusions

We saw a statistically significant association between companionship and lack of physical and verbal abuse subscore. Labour companionship was an under-implemented practice across the four countries, which was reflected in the low proportions of women who had a companion during labour and less so during birth. We interpret these findings by hypothesising that in situations of incomplete and rather sporadic implementation of companionship, effects may be limited. We call for policy advances to create sufficient space and support in terms of training and guidelines to support respectful treatment during childbirth.

## Supplementary material

10.1136/bmjph-2024-002462 online supplemental file 1

10.1136/bmjph-2024-002462 online supplemental file 6

10.1136/bmjph-2024-002462 online supplemental file 7

10.1136/bmjph-2024-002462 online supplemental file 8

## Data Availability

Data are available on reasonable request.
