# Supplementary material for: Labour companionship and respectful treatment of women during childbirth: a cross-sectional study across 16 hospitals in Benin, Malawi, Tanzania and Uganda
Source: BMJ Public Health. 2025 May 12;3(1):e002462. doi: 10.1136/bmjph-2024-002462 (PMC12086892; doi:10.1136/bmjph-2024-002462)
Supplement: online supplemental file 6 [file bmjph-3-1-s002.docx]

| **Subscore** | **Item** | | **Scale** | **Taken from** | **Changes to original tool** |
| --- | --- | --- | --- | --- | --- |
| **Maintained respect & dignity** | | Did the providers at the hospital treat you with respect? | 0=No, never, 1=Yes, a few times, 2=Yes, most of the time, 3=Yes, all of the time | Afulani et al. and Bohren et al. | Bohren al. used a different scale (strongly agree-strongly disagree), we followed Afulani's scale |
|  |  | Did the providers at the hospital treat you in a friendly manner? | 0=No, never, 1=Yes, a few times, 2=Yes, most of the time, 3=Yes, all of the time | Afulani et al. | we used "providers" instead of doctors, nurses or other staff |
|  |  | How would you describe the waiting time before you were admitted to the labour ward? | 0=Very short, 1=Just a little long, 2=Somewhat long, 3=Very long | Afulani et al. | question was reformulated from *How did you feel about the amount of time you waited? Would you say it was...* |
|  |  | Were you shouted or screamed at by a provider or other member of staff? | 0=No, 1=Yes, 2=Don't want to say, 3=Don't know | Bohren et al. | none |
|  |  | Were you mocked at by a provider or other member of staff? | 0=No, 1=Yes, 2=Don't want to say, 3=Don't know | Bohren et al. | none |
|  |  | Did you feel the providers at the hospital took the best care of you that they could? | 0=No, never, 1=Yes, a few times, 2=Yes, most of the time, 3=Yes, all of the time | Afulani et al. | none |
|  |  | Would you recommend a family member to give birth in the same hospital? | 1=Very strongly recommend, 2=Strongly recommend, 3=Recommend, 4=Undecided, 5=Do not recommend, 6=Strongly do not recommend, 7=Very strongly do not recommend | Bohren et al. | Bohren et al., include the following question "Overall, I am satisfied with the services I received during my stay at the hospital for childbirth" |
| **Maintained privacy and confidentiality** | | My private or personal information was shared without my consent | 0=No, never, 1=Yes, a few times, 2=Yes, most of the time, 3=Yes, all of the time | Bohren et al. | Reformulated from Bohren et al., (Did a staff member discuss your private information about your health in a way that others could hear?) |
|  |  | My physical privacy was violated e.g., being uncovered or having people in the delivery room without my consent | 0=No, never, 1=Yes, a few times, 2=Yes, most of the time, 3=Yes, all of the time | Bohren et al. | negative formulation compared to Bohren and different scale- I feel that my privacy was respected during examinations and treatments |
| **Lack of physical and verbal abuse** | | Were you slapped or pinched by a provider? | 0=No, 1=Yes, 2=Don't want to say, 3=Don't know | Bohren et al. | Two questions combined in 1 (pinched and slapped) |
|  |  | Did a provider make any negative comments e.g., about age/marital status/ethnicity/religion/HIV status? | 0=No, 1=Yes, 2=Don't want to say, 3=Don't know | Bohren et al. | options age, marital status, enthicity, religion and HIV are combined compared to Bohren |
|  |  | Were you shouted at or told off because you did not bring items with you? | 0=No, 1=Yes, 2=Don't want to say, 3=Don't know | neither | Both tools have a question on shouting but not particualrly for lack of items |
|  |  | Did any of the providers or other staff suggest or ask you (or your family) for a bribe, informal payment or gift? | 0=No, 1=Yes, 2=Don't want to say, 3=Don't know | Bohren et al. | none |
