# Supplementary material for: Labour companionship and respectful treatment of women during childbirth: a cross-sectional study across 16 hospitals in Benin, Malawi, Tanzania and Uganda
Source: BMJ Public Health. 2025 May 12;3(1):e002462. doi: 10.1136/bmjph-2024-002462 (PMC12086892; doi:10.1136/bmjph-2024-002462)
Supplement: online supplemental file 7 [file bmjph-3-1-s003.docx]

Selection of women for data collection


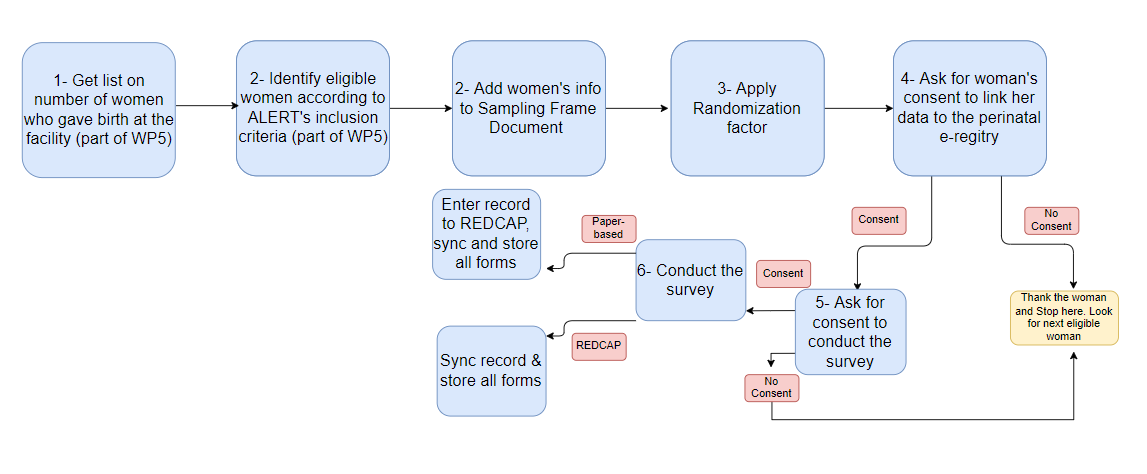


Table 2. Data collection dates per country and data collection round.

| **Country** | **Round 1 - Baseline** | **Round 2** | **Round 3** | **Round 4** | **Round 5** |
| --- | --- | --- | --- | --- | --- |
| **Benin** | 5^th^ December 2021 –  24^th^ January 2022 | 17^th^ June 2022 –  21^st^ July 2022 | 13^th^ December 2022 –31^st^ January 2023 | 25^th^ July 2023-  18^th^ August 2023 | Jan 31^st^ 2024 – feb 20^th^ 2024 |
| **Malawi** | 3^rd^ December 2021 –  22^nd^ December 2021 | 13^th^ August 2022 –  9^th^ September 2022 | 13^th^ February 2023 –  5^th^ March 2023 | 7^th^ August 2023-  26^th^ August 2023 | Feb 5^th^ 2024 – Feb 25^th^ 2024 |
| **Tanzania** | 9^th^ December 2021 –  22^nd^ December 2021 | 18^th^ July 2022 –  6^th^ August 2022 | 8^th^ March 2023 –  31^st^ March 2023 | 28^th^ August 2023-  20^th^ September 2023 | Feb 5^th^ 2024– Feb 29^th^ 2024 |
| **Uganda** | 28^th^ January 2022 –  16^th^ April 2022 | 8^th^ June 2022 –  11^th^ August 2022 | 17^th^ December 2022 –7^th^ April 2023 | 8^th^ June 2023-  July 14^th^ 2023 | 2^nd^ December 2023 – 8 January 2024 |
