# Supplementary material for: Labour companionship and respectful treatment of women during childbirth: a cross-sectional study across 16 hospitals in Benin, Malawi, Tanzania and Uganda
Source: BMJ Public Health. 2025 May 12;3(1):e002462. doi: 10.1136/bmjph-2024-002462 (PMC12086892; doi:10.1136/bmjph-2024-002462)
Supplement: online supplemental file 8 [file bmjph-3-1-s004.docx]

**Companionship coverage by data collection round on a country level**

**Benin**

|  | **Round 1**  **5 Dec 2021-24 Jan 2022** | **Round 2**  **17 Jun 2022-21 Jul 2022** | **Round 3**  **13 Dec 2022-31 Jan 2023** | **Round 4**  **25 Jul 2023-18 Aug 2023** | **Round 5**  **31 Jan 2024 – 20 Feb 2024** | **Total** |
| --- | --- | --- | --- | --- | --- | --- |
|  | *N= 201*  *n %* | *N= 203*  *n %* | *N= 202*  *n %* | *N= 196*  *n %* | *N= 201*  *n %* | *N=1003*  *n %* |
| **Present** | 29 (14.4) | 25 (12.3) | 19 (9.4) | 2 (1.0) | 23 (11.4) | 98 (9.8) |
| **Not present** | 155 (77.1) | 137 (67.5) | 135 (66.8) | 138 (70.4) | 178 (88.6) | 743 (74.1) |
| **Did not want** | 17 (8.5) | 41 (20.2) | 48 (23.8) | 56 (28.6) | 0 | 162 (16.1) |

**Malawi**

|  | **Round 1**  **3 Dec 2021 –22 Dec 2021** | **Round 2**  **13 Aug 2022 –9 Sep 2022** | **Round 3**  **13 Feb 2023 –5 Mar 2023** | **Round 4**  **7 Aug 2023-26 Aug 2023** | **Round 5**  **5 Feb 2024 – 25 Feb 2024** | **Total** |
| --- | --- | --- | --- | --- | --- | --- |
|  | *N= 214*  *n %* | *N=200*  *n %* | *N=200*  *n %* | *N=190*  *n %* | *N=200*  *n %* | *N=1004*  *n %* |
| **Present** | 68 (31.8) | 67 (33.5) | 93 (46.5) | 99 (52.1) | 130 (65.0) | 457 (45.5) |
| **Not present** | 138 (64.5) | 129 (64.5) | 106 (53.0) | 91 (47.9) | 70 (35.0) | 534 (53.2) |
| **Did not want** | 8 (3.7) | 4 (2.0) | 1 (0.5) | 0 | 0 | 13 (1.3) |

**Tanzania**

|  | **Round 1**  **9 Dec 2021 –22 Dec 2021** | **Round 2**  **18 Jul 2022–6 Aug 2022** | **Round 3**  **8 Mar 2023–31 Mar 2023** | **Round 4**  **28 Aug 2023-20 Sep 2023** | **Round 5**  **5 Feb 2024–29 Feb 2024** | **Total** |
| --- | --- | --- | --- | --- | --- | --- |
|  | *N=210*  *n %* | *N=204*  *n %* | *N= 203*  *n %* | *N= 203*  *n %* | *N= 176*  *n %* | *N=996*  *n %* |
| **Present** | 20 (9.5) | 18 (8.8) | 4 (1.9) | 4 (1.9) | 1 (0.6) | 47 (4.7) |
| **Not present** | 176 (83.8) | 186 (91.2) | 104 (51.2) | 191 (94.1) | 172 (97.7) | 829 (83.2) |
| **Did not want** | 14 (6.7) | 0 | 95 (46.8) | 8 (3.9) | 3 (1.7) | 120 (12.1) |

**Uganda**

|  | **Round 1**  **28 Jan 2022 –16 Apr 2022** | **Round 2**  **8 Jun 2022 – 11 Aug 2022** | **Round 3**  **17 Dec 2022 –7 Apr 2023** | **Round 4**  **8 Jun 2023-14 Jul 2023** | **Round 5**  **2 Dec 2023 – 8 Jan 2024** | **Total** |
| --- | --- | --- | --- | --- | --- | --- |
|  | *N=199*  *n %* | *N= 203*  *n %* | *N= 201*  *n %* | *N= 200*  *n %* | *N= 200*  *n %* | *N=1003*  *n %* |
| **Present** | 188 (94.5) | 198 (97.5) | 195 (97.0) | 194 (97.0) | 196 (98.0) | 971 (96.8) |
| **Not present** | 8 (4.0) | 5 (2.5) | 5 (2.5) | 6 (3.0) | 4 (2.0) | 28 (2.8) |
| **Did not want** | 3 (1.5) | 0 | 1 (0.5) | 0 | 0 | 4 (0.4) |
